# High resolution high density CMR circular tagging

**DOI:** 10.1186/1532-429X-15-S1-P88

**Published:** 2013-01-30

**Authors:** Ali Aghaeifar, Nafiseh Babaee, Abbas N Moghaddam

**Affiliations:** 1 Biomedical Engineering, Tehran Polytechnic, Tehran, Islamic Republic of Iran; 2 Electrical Engineering, Iran University of Science and Technology, Tehran, Islamic Republic of Iran; 3 Radiology, David Geffen School of Medicine at UCLA, Los Angeles, CA, USA

## Background

Wall thickening is the dominant factor for the effective ejection of the left ventricle (LV). Circular tagging is the suitable pattern for studying the radial strain which quantifies the wall thickening. High density tagging facilitates the post processing required for this quantification [1]. Following the recent sequence development for circular tagging, in this work we propose a k-spaced based approach for increasing the resolution and density of the circular tags during the reconstruction.

## Methods

In this study the polar HARP concept, which encodes the position of polar tags in the reconstructed phase, was extended using successive directional filters to decrease distortions that may appear in angle image. The density of taglines was then increased by multiplication of the phase of the angle images. The image resolution was also enhanced to accommodate the high density taglines. The following processes were all embedded in the online reconstruction of the images on the scanner: phase image extraction by polar harp, increasing the tag density through phase multiplication, zero-padding for increasing the spatial resolution, and finally masking the phase image based on the reconstructed magnitude image.

This study was conducted using ICE (Image Calculation Environment), which is the image reconstruction environment for Siemens scanners. ICE is built upon the C++ high-level programming language. The default reconstruction chain was modified and the entire process was embedded in a single functor in a separate pipeline. Total reconstruction time increased less than a fraction of a second for each frame.

The reconstruction chain was then examined on clinical Siemens scanners at 1.5T & 3.0T. Common MR parameters were set as following: 280 mm FOV, 6mm slice thickness, TE/TR= 2.89/52.24 ms, 250 Hz/pixel bandwidth, 15° flip angle, and 164x192 matrix size.

## Results

**Figure 1:**
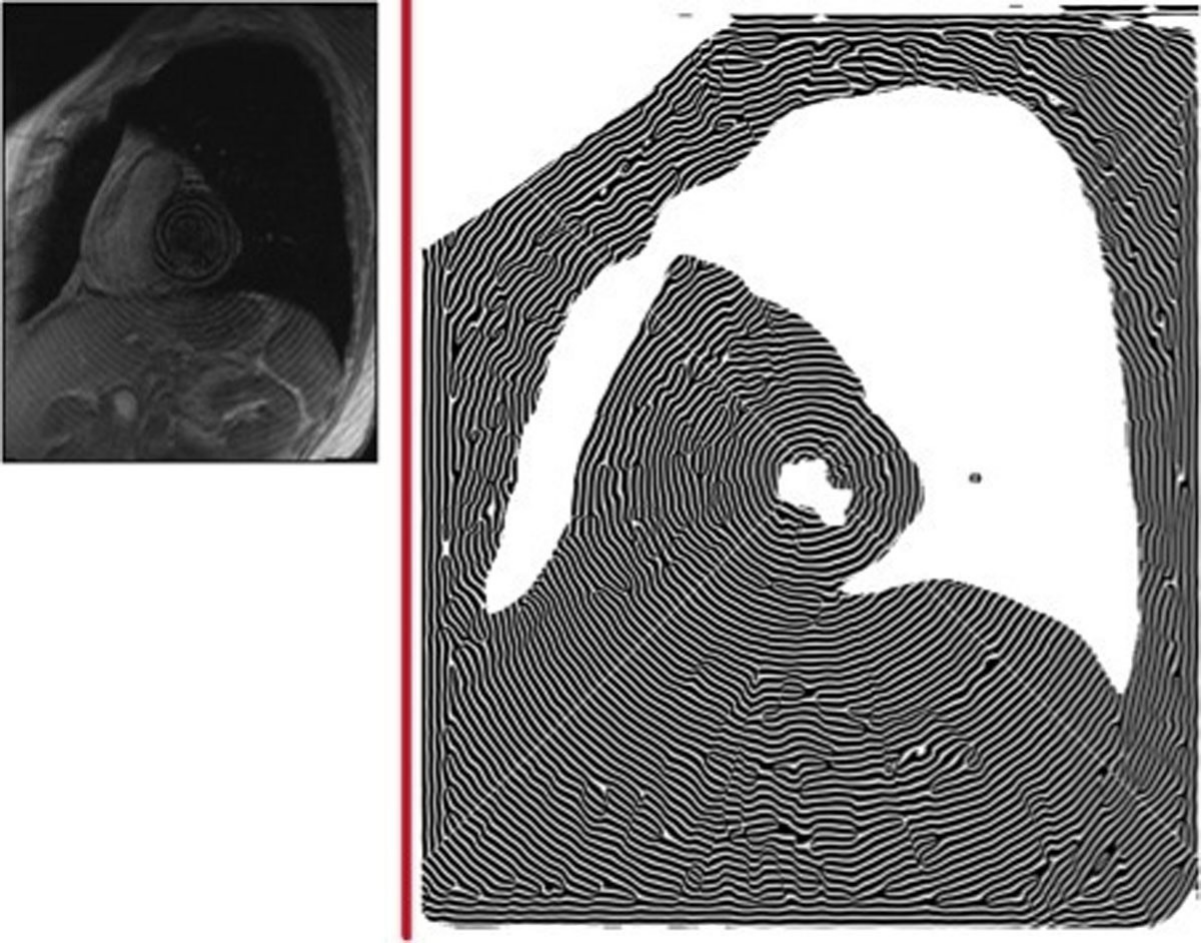


Figure 1 shows a short axis view of the myocardium in a healthy volunteer. The image size was primarily set to 164*192 pixels and after passing through inserted new functor enhanced to 656*768 pixels whereas the density of taglines was doubled. In practice these enhancements successfully examined up to 1640*1920 pixels. Since the taglines in the right image are reconstructed from the angle image, they have been synthesizes in regions such as blood pool. The fully synthesized taglines are highly distorted in these regions and can be filtered out.

## Conclusions

This work demonstrates the possibility of making high resolution high density CMR circular tagging on the myocardium. It further shows reconstruction pipeline has great potential for embedding offline processes. Since frequency and image domain both are accessible simultaneously in reconstruction chain, no further transform required and desired processes execute in almost real-time, which has wide clinical applications due to instant display of the images after acquisition.

## Funding

None

